# Complete Genome Sequence of Acinetobacter radioresistens Strain LH6, a Multidrug-Resistant Bacteriophage-Propagating Strain

**DOI:** 10.1128/MRA.00929-18

**Published:** 2018-08-09

**Authors:** Clay S. Crippen, Steven Huynh, William G. Miller, Craig T. Parker, Christine M. Szymanski

**Affiliations:** a Department of Microbiology and Complex Carbohydrate Research Center, University of Georgia, Athens, Georgia, USA; b Produce Safety and Microbiology Research Unit, Agricultural Research Service, U.S. Department of Agriculture, Albany, California, USA; University of Arizona

## Abstract

Antimicrobial resistance is a major problem worldwide. Understanding the interplay between drug-resistant pathogens, such as Acinetobacter baumannii and related species, potentially acting as environmental reservoirs is critical for preventing the spread of resistance determinants. Here we report the complete genome sequence of a multidrug-resistant bacteriophage-propagating strain of Acinetobacter radioresistens.

## Abstract (precis)

Antimicrobial resistance is a major problem worldwide. Understanding the interplay between drug-resistant pathogens, such as Acinetobacter baumannii and related species, potentially acting as environmental reservoirs is critical for preventing the spread of resistance determinants.

## ANNOUNCEMENT

Antibiotic resistance rates have increased in both hospital and community settings. Acinetobacter baumannii is considered a significant human health threat due to its pleiotropic survival strategies (1) and high propensity to develop antibiotic and biocide resistance. Fewer studies have been directed toward understanding the role of nonpathogenic species such as Acinetobacter radioresistens, which is the suspected origin of OXA-23 carbapenem resistance in A. baumannii (2) and has similar abilities to acquire antimicrobial resistance (AMR) determinants and survive extreme levels of oxidative stress, desiccation, and irradiation, suggesting that this species may play a role in AMR acquisition and spread among members of this genus (3, 4). We recently isolated the poultry commensal A. radioresistens strain LH6 and several bacteriophages capable of propagating on this strain (C. S. Crippen, R. T. Patry, M. J. Rothrock, Jr., S. Sanchez, and C. M. Szymanski, unpublished data). To further understand how LH6 could contribute to AMR spread, we report its genome sequence.

A single colony of strain LH6 was grown overnight to stationary phase, and the DNA was extracted as previously described (5). The genome was sequenced using the PacBio RS II next-generation sequencing platform and 20-kb SMRTbell libraries. PacBio reads were assembled using the hierarchical genome assembly process (HGAP) version 3.0 in the single-molecule real-time analysis package (v. 2.3.0). A final base call validation of the PacBio contig was performed using Illumina MiSeq 2 × 250-bp paired-end trimmed reads using a quality score threshold of ≥20 and the reference assembler within Geneious software v11.1. The final coverage for the PacBio contig was 182×. MiSeq base corrections required a minimum of 50× coverage and a MiSeq base call that was present in 75% of reads using the find variations job within Geneious. The final genome coverage was 378×.

A. radioresistens strain LH6 has a circular genome of 3,098,777 bp with an average GC content of 41.85%. The genome was annotated using the NCBI Prokaryotic Genome Annotation Pipeline (PGAP) and was generally confirmed by Rapid Annotation using Subsystem Technology (RAST) v. 2.0 (http://rast.nmpdr.org/) comparisons (6) with 2,756 putative protein-coding genes, 45 pseudogenes, 7 complete rRNA loci, and 76 tRNA genes predicted. Although LH6 is resistant to multiple antibiotic classes (Crippen et al., unpublished), no plasmids were identified in its genome. Also, no type I, II, or III restriction/modification systems were found, but LH6 encodes a type I-F CRISPR/Cas system and is predicted to contain at least two integrated prophages (7).

PGAP annotation indicates that strain LH6 encodes many mechanisms for virulence and persistence, including enzymes for metal use and resistance (e.g., copper, zinc, cobalt, and cadmium), detoxification (arsenic), and efflux of quaternary ammonia compounds (QacE) (8). We also identified several putative multidrug efflux systems, a chloramphenicol acetyltransferase, β-lactamase enzymes, and pathways for fluoroquinolone resistance, in addition to proteins involved in resistance to oxidative stress, carbon starvation, detoxification of radical oxygen species, osmotic stress tolerance, type VI secretion, and colicin V export (CvpA) (9). We identified a capsule/exopolysaccharide biosynthesis gene cluster encoding homologs of Wza, Wzb, and Wzc and genes predicted to encode enzymes involved in lipooligosaccharide and trehalose biogenesis. All these features indicate that this strain is multidrug resistant, environmentally resilient, and capable of multiple mechanisms of genetic exchange, making LH6 an ideal reservoir for AMR spread.

### Data availability.

The genome sequence of A. radioresistens strain LH6 has been deposited in GenBank under the accession number CP030031. The sequencing reads have been deposited under the accession numbers SRR7533604 and SRR7533605. All reads have been deposited to SRA and are associated with BioProject number PRJNA475995.

## References

[1] Xie R, Zhang XD, Zhao Q, Peng B, Zheng J 2018 Analysis of global prevalence of antibiotic resistance in *Acinetobacter baumannii* infections disclosed a faster increase in OECD countries. Emerg Microbes Infect 7:31. doi:10.1038/s41426-018-0038-9. 29535298 PMC5849731

[2] Poirel L, Figueiredo S, Cattoir V, Carattoli A, Nordmann P 2008 *Acinetobacter radioresistens* as a silent source of carbapenem resistance for *Acinetobacter* spp. Antimicrob Agents Chemother 52:1252–1256. doi:10.1128/AAC.01304-07. 18195058 PMC2292503

[3] Jawad A, Snelling AM, Heritage J, Hawkey PM 1998 Exceptional desiccation tolerance of *Acinetobacter radioresistens*. J Hospital Infect 39:235–240. doi:10.1016/S0195-6701(98)90263-8. 9699144

[4] McCoy KB, Derecho I, Wong T, Tran HM, Huynh TD, La Duc MT, Venkateswaran K, Mogul R 2012 Insights into the extremotolerance of *Acinetobacter radioresistens* 50v1, a Gram-negative bacterium isolated from the Mars Odyssey spacecraft. Astrobiology 12:854–862. doi:10.1089/ast.2012.0835. 22917036

[5] Miller WG, Pearson BM, Wells JM, Parker CT, Kapitonov VV, Mandrell RE 2005 Diversity within the *Campylobacter jejuni* type I restriction-modification loci. Microbiology 151:337–351. doi:10.1099/mic.0.27327-0. 15699185

[6] Aziz RK, Bartels D, Best AA, DeJongh M, Disz T, Edwards RA, Formsma K, Gerdes S, Glass EM, Kubal M, Meyer F, Olsen GJ, Olson R, Osterman AL, Overbeek RA, McNeil LK, Paarmann D, Paczian T, Parrello B, Pusch GD, Reich C, Stevens R, Vassieva O, Vonstein V, Wilke A, Zagnitko O 2008 The RAST server: Rapid Annotations using Subsystems Technology. BMC Genomics 9:75. doi:10.1186/1471-2164-9-75. 18261238 PMC2265698

[7] Touchon M, Cury J, Yoon EJ, Krizova L, Cerqueira GC, Murphy C, Feldgarden M, Wortman J, Clermont D, Lambert T, Grillot-Courvalin C, Nemec A, Courvalin P, Rocha EP 2014 The genomic diversification of the whole *Acinetobacter* genus: origins, mechanisms, and consequences. Genome Biol Evol 6:2866–2882. doi:10.1093/gbe/evu225. 25313016 PMC4224351

[8] Liu WJ, Fu L, Huang M, Zhang JP, Wu Y, Zhou YS, Zeng J, Wang GX 2017 Frequency of antiseptic resistance genes and reduced susceptibility to biocides in carbapenem-resistant *Acinetobacter baumannii*. J Med Microbiol 66:13–17. doi:10.1099/jmm.0.000403. 27930267

[9] Cohen LJ, Han S, Huang YH, Brady SF 2018 Identification of the colicin V bacteriocin gene cluster by functional screening of a human microbiome metagenomic library. ACS Infect Dis 4:27–32. doi:10.1021/acsinfecdis.7b00081. 28810737 PMC6592431

